# An assessment of prevalence and expenditure associated with discharge brain MRI in preterm infants

**DOI:** 10.1371/journal.pone.0247857

**Published:** 2021-03-05

**Authors:** Keith A. Dookeran, James M. Groh, David G. Ritacco, Lydia R. Marcus, Yang Wang, Janine Y. Khan

**Affiliations:** 1 Joseph J. Zilber School of Public Health, University of Wisconsin-Milwaukee, Milwaukee, Wisconsin, United States of America; 2 School of Public Health, Division of Epidemiology and Biostatistics, University of Illinois at Chicago, Chicago, Illinois, United States of America; 3 The Cancer Foundation for Minority and Underserved Populations, Chicago, Illinois, United States of America; 4 Marion County Public Health Department, Epidemiology, Indianapolis, Indiana, United States of America; 5 Department of Pediatrics, Division of Neurology, Ann & Robert H. Lurie Children’s Hospital, Northwestern University, Chicago, Illinois, United States of America; 6 Department of Pediatrics, Division of Neonatology, Ann & Robert H. Lurie Children’s Hospital, Northwestern University, Chicago, Illinois, United States of America; Hopital Robert Debre, FRANCE

## Abstract

To assess national expenditure associated with preterm-infant brain MRI and potential impact of reduction per *Choosing Wisely* campaign 2015 recommendation to “avoid routine screening term-equivalent or discharge brain MRIs in preterm-infants”. Cross-sectional U.S. trend data from the *Agency for Healthcare Research and Quality* (AHRQ), *Healthcare Cost and Utilization Project* (HCUP) *Kids’ Inpatient Database* (KID) database (2006, 2009, 2012, 2016) was used to estimate overall national expenditure associated with brain MRI among infants with gestational age (GA) ≤36 weeks, and also when classified as ‘not indicated’ (NI-MRI) i.e., equivalent to routine use without clinical indications and regarded as low-value service (LVS). Associated cost was determined by querying CMS-database for physician-fee-schedules to find the highest global procedure-cost per cycle, then adjusting for inflation. Sensitivity-analyses were conducted to account for additional clinical charges associated with NI-MRI. 3,768 (0.26%) of 1,472,236 preterm-infants had brain MRI across all cycles (inflation-adjusted total $3,690,088). Overall proportion of brain MRIs increased across 2006–2012 from 0.25%-0.33% but decreased in 2016 to 0.16% (*P*<0.001). Inflation-adjusted overall expenditure by cycle was: 2006, $1,299,130 (95% CI: $987,505, $1,610,755); 2009, $1,194,208 (95% CI: $873,487, $1,516,154); 2012, $931,836 (95% CI: $666,114, $1,197,156); and 2016, $264,648 (95% CI: $172,061, $357,280). Prevalence for NI-MRI in 2006, 2009, 2012 and 2016 was 86% (n = 809), 88% (n = 940), 89% (n = 1028) and 50% (n = 299), respectively; and 70% were in infants 35–36 weeks GA. NI-MRI prevalence was not different over time by payer-type (Medicaid, private), sex or race/ethnicity (white, black, Hispanic); larger hospital size was significantly associated across 2006–2012 but this declined for all sizes in 2016, with most decline in larger hospitals (*P* for interaction <0.05). NI-MRI expenditure sensitivity-analysis with addition of cycle median total-admission-charge to inflation-adjusted CMS-fee was $1,190,919/$518,343, for 2012/2016 cycles respectively. National MRI prevalence in preterm infants (both overall and LVS) and associated expenditure decreased substantially post recommendation; however, annual savings are modest and unlikely to be >$1.2 million.

## Introduction

In 2011 Berwick and Hackbarth estimated that overuse of medical services accounted for roughly $200 billion in wasteful spending in the United States [1]. This led to the *American Board of Internal Medicine* (ABIM) Foundation, Consumer Reports, and nine medical specialty societies launching the Choosing Wisely campaign in 2012, that was motivated by the idea that health care professionals and specialty societies should take the lead in defining when to avoid treatments and tests that are unnecessary or harmful—that is, low-value care [2, 3].

In an effort to reduce wasteful spending in perinatal care, the *American Academy of Pediatrics* (AAP) Section on Perinatal Pediatrics created a *Top Five* list of low value services (LVS) in newborn medicine [4] as part of the *Choosing Wisely* campaign [5]. This LVS list targets tests and treatments determined to be unjustified based on efficacy, safety, or cost, and decreased use would therefore suggest a meaningful reduction in wasteful spending [4].

The *Top Five* list includes a recommendation to “avoid routine screening term-equivalent or discharge brain MRIs in preterm infants” on the basis that there is “insufficient evidence that the routine use of term-equivalent or discharge screening brain MRIs in preterm infants improves long-term outcome” [4, 6, 7]; as such, routine MRI in this scenario can be regarded as synonymous with LVS and not being clinically indicated. It is also likely that brain MRIs in preterm infants further increase wasteful spending due to costs associated with additional clinical investigation related to incidental findings [8, 9]. Ho et al. state that “roughly $60 million or more (is) expended annually for brain MRIs in the ~ 60,000 very low birth weight infants born each year in the US” [6].

The actual national prevalence and expenditure for brain MRI, however, whether overall, or not indicated (i.e., equivalent to routine use without clinical indications and regarded as LVS) in this clinical context is unclear. We chose to further study because this recommendation is associated with the most expensive LVS procedure identified on the *Top Five* list and could potentially substantiate tens of millions of dollars reduction in wasteful spending [4, 6, 7]. Our objectives were: (1) to assess national prevalence of preterm infant brain MRI and associated expenditure and impact of reduction per *Choosing Wisely* campaign recommendation; and (2) to examine factors that may be associated with LVS MRI prevalence.

## Materials and methods

Our study utilized data from the most recent *Agency for Healthcare Research and Quality* (AHRQ), *Healthcare Cost and Utilization Project* (HCUP) *Kids’ Inpatient Database* (KID). The HCUP-KID database is a nationally representative sample of pediatric inpatient discharges; it captures newborn/perinatal care and has been produced almost every three years since 1997. Per AHRQ guidance this study utilized KID database statistical discharge weights to obtain national estimates [10]. Our study used data available from 2006, 2009, 2012 and 2016 cycles, which when weighted, estimates roughly 7 million hospitalizations per year [11].

Brain MRIs among preterm infants were identified using available *International Classification of Diseases*, *Ninth and Tenth Revision* (ICD-9 and ICD-10) diagnosis and procedure codes [12]. Inclusion/exclusion criteria: ICD-9/10 diagnosis codes were used to choose preterm infants with gestational age (GA) ≤ 36 weeks (ICD-9: *76*.*521*, *76*.*522*, *76*.*523*, *76*.*524*, *76*.*525*, *76*.*526*, *76*.*527*, *76*.*528*; ICD-10: *P07*.*22*, *P07*.*33*, *P07*.*35*, *P07*.*36*, *P07*.*37*, *P07*.*38*, *P07*.*39*), among which we then restricted our analysis to those who received a brain MRI using ICD-9 procedure code *88*.*91* (2006–2012) and ICD-10 procedure code *B030Y0Z* (2016). Our dataset consisted of 1,472,236 preterm infants from 2006, 2009, 2012, and 2016, and the extent of missing data was < 5% for all variables, except for race which was 10.2%.

We first examined overall national prevalence of preterm infant brain MRI and associated expenditure. Next we evaluated LVS MRI prevalence and the impact of reduction per recommendation; for this analysis preterm infants who received brain MRIs were clinically classified as “indicated” or “not indicated” (NI-MRI) using an assessment of procedure appropriateness per diagnosis as follows. Not indicated imaging was assigned to routine screening term-equivalent (TE) MRI performed on preterm infants who are at risk for adverse neurodevelopmental outcome, without definitive clinical and/or cranial ultrasound findings that predispose to underlying brain abnormality, whereas indicated imaging was assigned to MRI performed in response to clinical scenarios and/or cranial ultrasound findings that raised concern for neurologic abnormality [13]. A senior neonatologist (JYK) and senior pediatric neurologist (DGR) independently reviewed all principal clinical diagnoses (i.e., KID data elements: DXn and I10_DXn *first listed diagnoses* are the principal diagnoses defined as conditions established after study to be chiefly responsible for occasioning the admission of the patient to the hospital for care) with accompanying ICD-9/10 diagnosis codes in the selected preterm dataset for appropriateness of MRI procedure indication. A preterm infant who received a brain MRI was then classified as “indicated” vs. “non-indicated” by both physicians independently (i.e., physicians performed classification in a blinded manner). Code discrepancies were subsequently reconciled via discussion and consensus by the physicians and the final list of indicated/non-indicated diagnoses is provided (see S1 Table). A kappa-statistic (measure of inter-rater agreement) was calculated for ICD-9 and ICD-10 diagnoses classified as NI-MRI. This was found to be 0.935 for ICD-9 and 0.9078 for ICD-10 (both *P* <0.001). These values indicated substantial agreement between raters prior to code discrepancy reconciliation.

In consultation with clinical neonatologists and neurologists, we performed a contextually informed systematic review of the KID data elements dictionary and included in our analysis all covariates related to preterm infant brain MRI use and available across the database cycles [14]: sex; payer type (Medicaid/private); race/ethnicity (black/white/Hispanic); hospital size [(large/medium/small); per KID documentation—beginning in 2000, the hospital’s bed size categories are defined using region of the U.S., the urban-rural designation of the hospital, in addition to the teaching status [15]]; hospital type by teaching status (urban non-teaching/urban teaching); hospital geographic region (Northeast/Midwest/South/West); total charges ($1000s); length of stay (days); and cycle year [15].

Overall expenditure was assessed using: (1) total admission charges from KID [the ‘total charges’ covariate used is the cleaned KID data element and generally does not include professional fees and non-covered charges which are removed from the charge during HCUP processing]; and (2) claims charges based on inflation-corrected fees for brain MRI, determined by querying CMS online database for physician fee schedules to find the highest global procedure cost per cycle for brain MRI [16] [the ‘highest CMS global procedure cost’ (i.e., both the technical and professional components of the procedure) for the non-facility limiting charge are selected as this is the maximum amount a beneficiary can be charged for the service, and generally Medicare provides higher payments to physicians and other health care professionals for procedures performed in their offices because they are responsible for providing clinical staff, supplies, and equipment] for relevant years [17]. First, baseline CMS fee cost estimates are developed for each cycle, specifically: 2007 (as 2006 data not available), $1120; 2009, $932; 2012, $720; 2016, $415. Next, these estimates are corrected for inflation using an inflation calculator [18] standardizing to 2020 cost, specifically: 2007, $1385; 2009, $1114; 2012, $804; 2016, $443. Sensitivity analyses were conducted to account for additional charges associated with NI-MRI (per need for additional clinical investigation/ workup) using a combination of these measures (i.e., by adding 10%, 25% and 50% of the total admission charge per cycle, from the KID data to the inflation-corrected CMS fees, and then comparing cost estimates). We assessed utilization trends across cycles by measuring prevalence of all preterm infant brain MRI and, by indicated vs NI-MRI status, and then estimating associated expenditure.

We examined covariate associations by NI-MRI status. Multivariable generalized linear regression models were used to estimate associations with outcomes of NI-MRI (vs. indicated) and total charges (continuous value), overall and across cycles (predictive margins) and reported as risk differences (RDs) with 95% confidence intervals (CIs). Interaction terms between time (cycle/year) and covariates were included in fully specified multivariable models and final models for trend in mean NI-MRI or total charges across cycles were adjusted for sex, race, hospital size, hospital type, hospital region, and payer type. All analyses were conducted using Stata statistical software, version 16 (Stata Corp., College Station, TX), and account for complex survey design, and a common primary sampling unit survey weight variable was created across KID database cycles for this analysis. All tests are two-sided with a threshold for significance of 0.05.

Our study used deidentified admission claims data from a public registry and, after review by University of Wisconsin-Milwaukee Institutional Review Board, was deemed to not involve research with human subjects.

## Results

Our dataset was representative of 1,472,236 preterm infants from 2006, 2009, 2012, and 2016 of which 0.26% (n = 3,768; 95% CI: 0.248, 0.264) received brain MRIs. Table 1 shows the distribution of study baseline characteristics by LVS status (i.e., non-indicated vs. indicated status). The prevalence of preterm infants who received brain MRIs increased marginally from 2006 to 2012 [2006: n = 938 (0.25%: 95% CI: 0.235, 0.268); 2009: n = 1,072 (0.29%: 95% CI: 0.270, 0.304); 2012: n = 1159 (0.33%: 95% CI: 0.313, 0.352)], but significantly decreased in 2016 to 0.16%: (n = 597; 95% CI: 0.144, 0.169). Across all years, 82% were NI-MRI (n = 3076) and 18% were indicated (n = 692). The proportion of NI-MRI remained relatively constant across 2006–2012 [86% in 2006 (n = 809), 87% in 2009 (n = 940) and 89% 2012 (n = 1028)] but declined significantly to 50% in 2016 (n = 299; *P* for trend <0.001).

**Table 1:** Distribution of population characteristics by MRI indication status.

	Total (N = 3,768)	Indicated Infant (n = 692)	Non-Indicated Infant (n = 3,076)	p-value
	N	N (W%^a^)	N (W%^a^)	
Sex				0.86
Male	2,064	382 (55.2)	1,683 (54.8)	
Female	1,701	310 (44.8)	1,055 (44.6)	
Race/Ethnicity				0.53
White	1,269	247 (40.3)	1,022 (37.0)	
Black	630	114 (18.6)	516 (18.7)	
Hispanic	912	156 (25.4)	756 (27.4)	
Payer Type				0.72
Medicaid	2,057	387 (56.0)	1,670 (54.3)	
Private	1,401	246 (35.6)	1,155 (37.6)	
Hospital Size				<0.001
Small	263	95 (14.4)	169 (5.7)	
Medium	673	157 (23.9)	516 (17.3)	
Large	2,694	406 (61.7)	2,288 (77.0)	
Hospital Type				0.30
Urban non-teaching	370	49 (9.6)	321 (14.0)	
Urban teaching	2,446	468 (90.4)	1,978 (86.1)	
Hospital Region				0.01
Northeast	989	129 (18.6)	861 (28.0)	
Midwest	569	125 (18.1)	444 (14.4)	
South	981	246 (35.6)	735 (23.9)	
West	1,227	192 (27.7)	1,036 (33.7)	
Year				<0.001
2006	938	129 (18.7)	809 (26.3)	
2009	1,072	133 (19.2)	940 (30.6)	
2012	1,159	131 (18.9)	1,028 (33.4)	
2016	597	299 (43.3)	299 (9.7)	
Mean Total Charge ($1,000) (SE)	442.3 (30.6)	326.6 (39.9)	468.8 (33.7)	<0.001

^a^Weighted percentages, weighted to total discharged population in AHA universe.

There were no differences in distribution of NI-MRI among preterm infants based on sex, payer type, race/ethnicity, and hospital type. However, significant differences were observed based on hospital size and region, such that NI-MRI were more likely to be performed in large hospitals and in the Northeast and West (all *P* <0.001).

**Fig 1:**
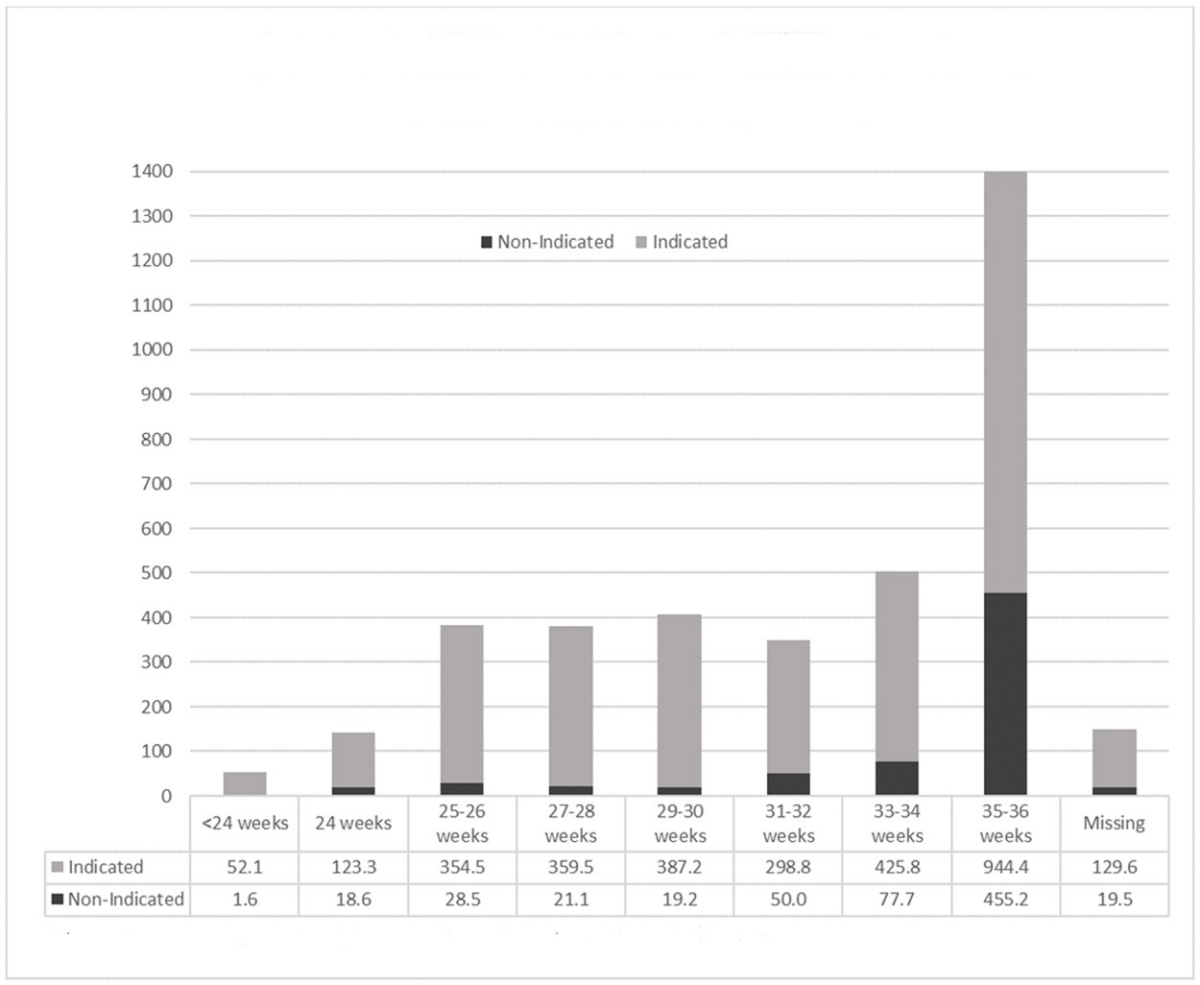
Distribution infants across gestational age and by MRI indication status. ^a^ values are weighted absolute numbers of indicated and non-indicated infants.

Fig 1 shows the absolute prevalence of infants across GA and the study sample was right skewed with most observations in the 35–36 weeks GA. In addition, 70% of NI-MRI occurred in infants 35–36 weeks GA (S1 Fig). Results of multivariable modeling of risk factors associated with non-indicated brain MRI status are provided in Table 2 below.

**Table 2:** Multivariable generalized linear regression of risk factors for non-indicated brain MRIs^1^.

Covariate	Coefficient	SE	95% CI
Female [Reference: Male]	0.008	0.017	(-0.025, 0.042)
Race [Reference: White]			
Black	0.042	0.021	(0.000, 0.084)
Hispanic	0.022	0.024	(-0.025, 0.068)
Hospital Size [Reference: Small]			
Medium	0.089	0.068	(-0.044, 0.223)
Large	0.148	0.056	(0.038, 0.259)
Hospital Type [Reference: Urban, nonteaching]			
Urban, teaching	-0.010	0.028	(-0.065, 0.044)
Primary Payer [Reference: Medicaid]			
Private insurance	0.004	0.022	(-0.039, 0.046)
Year [Reference: 2006]			
2009	0.045	0.029	(-0.011, 0.102)
2012	0.026	0.030	(-0.034, 0.085)
2016	-0.367	0.052	(-0.469, -0.265)

^1^Survey weights used, weighted to total discharged population in AHA universe; model F-test: p< 0.0001; model goodness of fit: p = 0.081

^2^Analyses were re-run with product terms. Results are demonstrated in the marginal plots shown in Fig 2.

**Fig 2:**
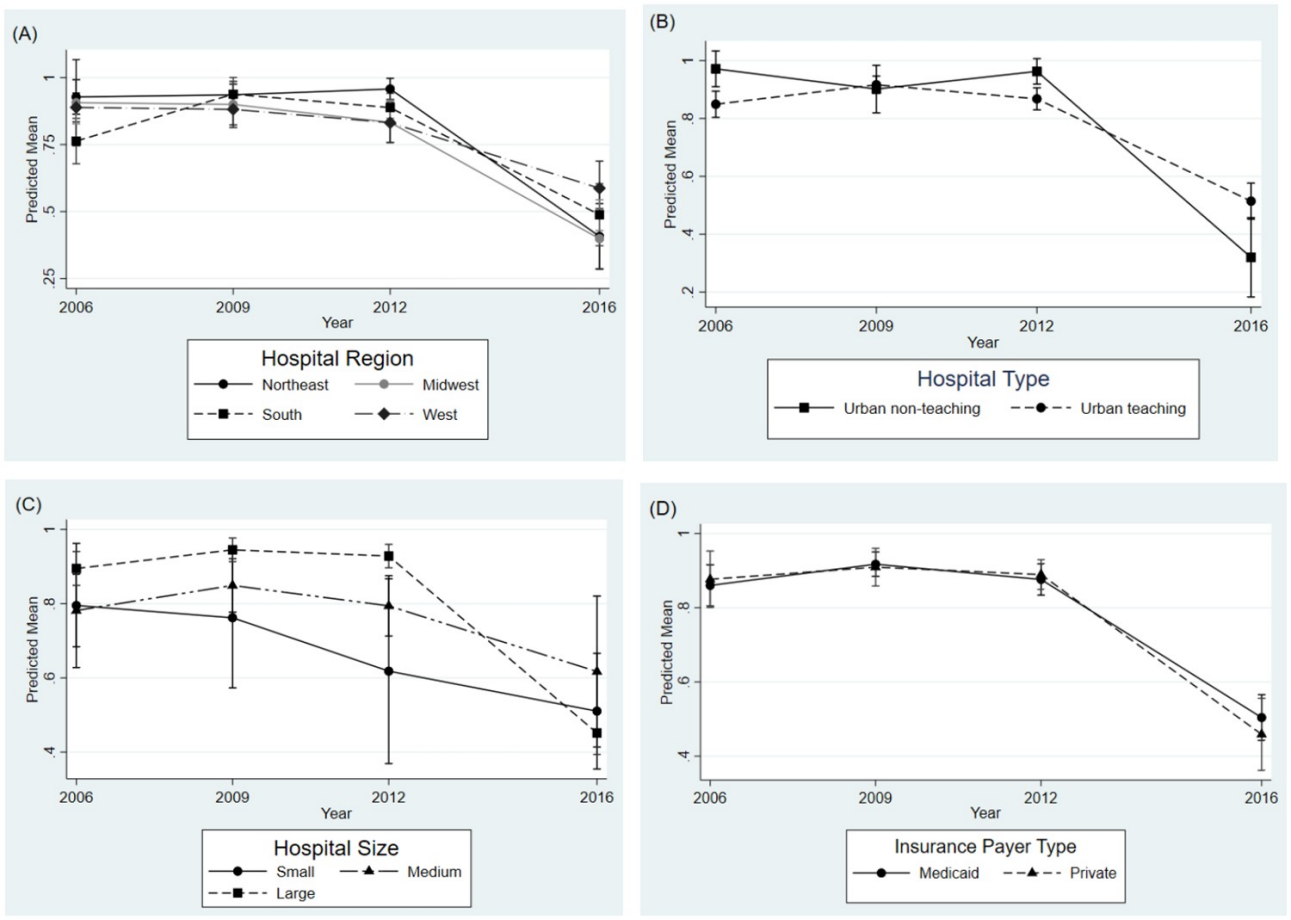
Trends in LVS non-indicated MRI across cycles. Panel A: Stratified by Hospital Region. Panel B: Stratified by Hospital Type. Panel C: Stratified by Hospital Size. Panel D: Stratified by Insurance Payer Type.

Fig 2 shows trends in LVS NI-MRI across cycles stratified by specific hospital/ insurance factors. Hospital size, region and type demonstrated interaction with cycle (*P* for interaction <0.05), while payer status did not. Hospitals in the Northeast had the highest NI-MRI rates from 2006–2012 but decreased to be among the lowest in 2016 (*Panel A*). Urban non-teaching vs. teaching hospitals were seen to have higher NI-MRI rates across 2006–2012, but both substantially decreased in 2016, with a larger decline in urban non-teaching (*Panel B*). Larger and medium hospital size compared with small size was significantly associated with NI-MRI across 2006–2012, but declined for all sizes in 2016, with the largest decline observed in larger hospitals (*Panel C*). In addition, when GA was dichotomized as < 35 vs. 35–36 weeks, the prevalence of MRI overall and NI-MRI (*P* for trend <0.05) were observed to decrease in the 2016 cycle among both subgroups compared with prior cycles (S2 Table).

**Fig 3:**
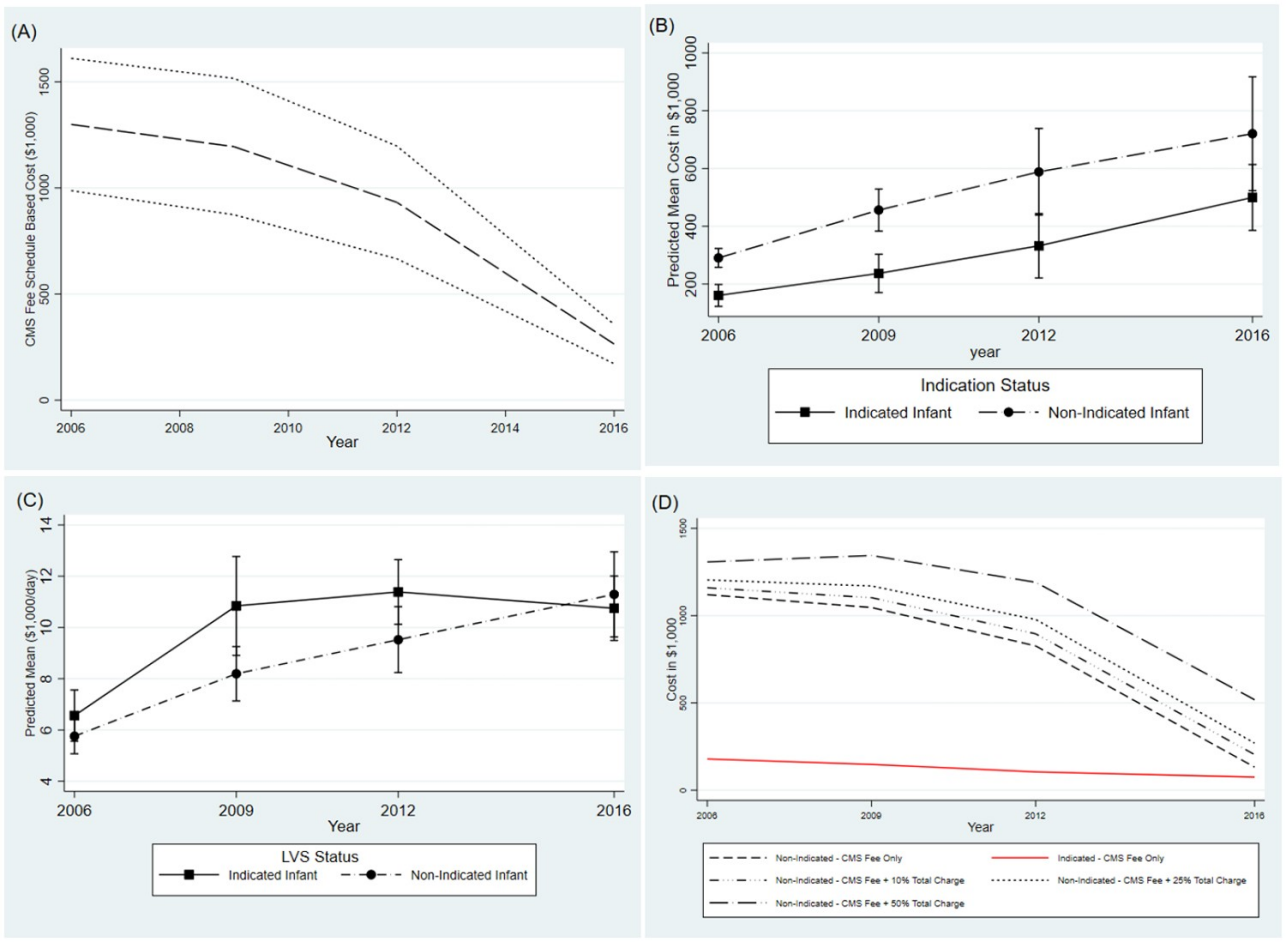
Trends in cost associated with preterm brain MRI across cycles. Panel A: Total Inflation Corrected CMS Fee Schedule Based Cost with 95% CIs for Preterm Brain MRI by KID cycle. Panel B: Unadjusted Mean Total Charge across cycles by Indication Status. Panel C: Length of Stay Adjusted Mean Total Charge across cycles by Indication Status. Panel D: Sensitivity Analysis for Additional Cost Related to Preterm Brain MRI.

Fig 3 shows trends in cost associated with preterm brain MRI across cycles. Total estimated CMS fee-schedule-based cost for preterm brain MRI was $3,690,088 (inflation adjusted) for all cycles, but this cost declined with each successive KID Cycle. Inflation adjusted overall expenditure by cycle was: 2006, $1,299,130 (95% CI: $987,505, $1,610,755); 2009, $1,194,208 (95% CI: $873,487, $1,516,154); 2012, $931,836 (95% CI: $666,114, $1,197,156); and 2016, $264,648 (95% CI: $172,061, $357,280) (*Panel A*). Crude total charges for NI-MRI vs. indicated based on KID data was significantly higher overall ($469,000 vs. $327,000; *P* <0.001), and trends for total charges across cycles demonstrate almost linear increase over time (*Panel B*). However, when KID data total charge was adjusted for length of stay, NI-MRI total charges were lower across cycles and this difference was not found to be statistically significant (*Panel C*). Sensitivity analyses with addition of 10%, 25% and 50% of the total admission charge from the KID data to the inflation adjusted CMS fees show cost associated with NI-MRI in 2012 to be $896,222, $978.238, and $1,190,919, respectively; but decreased in 2016 to $204,221, $270,036, and $518,343, respectively (*Panel D*). Hence, sensitivity analysis suggests a potential range of annual savings of between $518,343 to $1,190,919 for reduction of LVS/routine NI-MRI based on 2012 and 2016 cycles as most recent data.

## Discussion

National health spending in the U.S., accounting for approximately 18% of Gross Domestic Product (GDP), has been increasing at an alarming rate [19]. Recent CDC data show that U.S. preterm birth rate (i.e. births at less than 37 weeks GA) rose from 9.57% to 9.85% during 2014–2016 [20], and 2018 data show 10% and 8.3% of 3,791,712 total births are described as preterm and low birth weight (i.e., < 2500 grams), respectively [21]. Ho et al. suggest that roughly $60 million or more is expended annually for brain MRIs in approximately 60,000 very low birth weight infants born each year in the U.S., and it is likely that incidental findings on MRI further increase waste due to costs associated with need for additional clinical investigation [4–6, 8, 9]. Overall, these data suggest high prevalence of LVS/routine NI-MRI practice and substantial wasteful spending.

Recently, Tolia et al. demonstrated the pattern of neuroimaging among infants ≤ 33 weeks between 2008 to 2017 [22]. They showed that the number of infants investigated with any neuroimaging modality declined during the 10-year period, driven primarily by a reduction in cranial ultrasound in infants born at 31–33 weeks gestational age; however, MRI use in those ≤ 33 weeks increased until 2015, and then decreased [22].

Similarly, our study findings indicate that following implementation of *Choosing Wisely* in newborn medicine in 2015, national MRI prevalence in preterm infants (both overall and LVS) and associated expenditure decreased substantially post recommendation. Importantly, compared with 2006–2012 cycles, the 2016 cycle shows a substantial decrease in the number of LVS NI-MRIs in preterm infants (>86% vs. 50%, respectively). This suggests that the recommendations to not perform so many non‐indicated MRI’s was likely clinically embraced. Overall, we also show that the national prevalence of MRIs is low, affecting only 0.26% of all preterm infants over the four cycles. In addition, both the prevalence (0.16%) and calculated reimbursement for MRI’s overall also significantly decreased in 2016. Hence, the cost-savings associated with this *Top Five* guideline is modest; if all LVS NI-MRIs between 2012–2016 were eliminated, nationwide savings would be estimated maximally at less than $5 million (using sensitivity analysis with addition of cycle median total admission charge value); it should be noted that these estimates are substantially lower than those previously stated by Ho et al. [6]. We are confident that this estimate would account for additional costs associated with further workup due to incidental findings as recent reports indicate prevalence of incidental findings on routine brain MRI in preterm infants at approximately 10% [8, 9].

Further, while reduction of NI-MRI would reduce excess healthcare spending, the relative reduction would have a minimal impact on wasteful spending in children younger than one-year which totaled $50.4 billion in 2013 alone [23]; and according to the U.S. Bureau of Labor Statistics, prices for medical care were 17.2% higher in 2019 compared with 2013, which would predict expected expenditure in this group to be closer to $59.1 billion [24]. Interestingly, we found that large hospitals, those in the northeast, and urban non-teaching hospitals had higher use of NI-MRI up to 2012, but this decreased significantly in 2016 and may be credited to *Choosing Wisely*.

Ho et al. state that “studies must show that term-equivalent brain MRIs are superior to neurodevelopmental evaluations, or less expensive than more accessible technologies like intracranial ultrasound, to warrant routine use” [6]; these considerations highlight the importance of related procedure efficacy and costs. However, considering the modest cost-savings for this specific LVS, from a public health and value-based care perspective, we advocate that for true waste reduction to occur there must also be some consideration of procedure prevalence and estimates projected for associated cost-savings from reduction. Recently, Kerr et al. advocated that for the *Choosing Wisely* campaign to have a meaningful impact on wasteful healthcare spending, the LVS identified in the *Top Five* lists must be prioritized and evaluated [3]; and our study contributes to such evaluation. As in the case of the *Top Five* list created by the AAP *Section on Perinatal Pediatrics*, many of the guidelines generated as part of the *Choosing Wisely* campaign, were chosen by practicing physicians in their respective field, without the inclusion of public health professionals or health-economists [4]. As such, many of the LVS included within the *Top Five* lists are heuristic-based procedures and practices that may be infrequently used by health providers across the country.

National health spending in the U.S. is projected to reach $6.2 trillion by 2028 [19, 25]. Recently, Shrank et al. estimated that the cost of waste in the U.S. health care system ranged from $760 billion to $935 billion, accounting for approximately 25% of total health care spending, and the projected potential savings from interventions that reduce waste, excluding savings from administrative complexity, ranged from $191 billion to $286 billion, representing a potential 25% reduction in the total cost of waste; this highlights the need for implementation of effective measures to eliminate waste and represents an opportunity to reduce the continued increases in US health care expenditures [25, 26]. Wasteful spending is multifaceted and may arise from the failure to deliver care [27], failure to coordinate care [28], overuse of tests, overtreatment [3], administrative structures [29], regulation, and/or overpricing [30]. As such, a multipronged approach is needed to effectively address this growing problem.

We acknowledge that performance of evidence‐based indicated clinical procedures is important, and that non‐indicated MRI’s are stressful to infants and staff, frequently necessitating sedation and close monitoring, and consuming valuable nursing and physician time as well as equipment costs. Our study is the first to evaluate the prevalence and cost-savings of reducing LVS brain NI-MRI among preterm infants in neonatal medicine. Screening TE MRI was previously recommended for extremely preterm infants who are at risk for adverse neurodevelopmental outcome, including cerebral palsy, cognitive and language deficits, and neurosensory impairment, depending on the region, as well as extent of perinatal and/or neonatal brain injury [13]. The *Choosing Wisely* initiative aimed to discourage the routine use of TE MRI screening in preterm infants because there is no evidence that it improves outcome as it cannot predict cognitive and language outcome, or increased risk of autism, and does little to allay parental anxiety about future neurodevelopmental outcomes [31]. While it is true that utilization of this screening modality will not, in itself, improve outcome, it nevertheless may inform the direction and intensity of rehabilitative services by providing actionable information, before deficits are appreciated through neurological and developmental assessment during follow-up clinical visits months after discharge [32]. Regarding the human brain, there are still many unanswered questions and the literature and value of TE MRI continues to evolve. A survey of members of the Newborn Brain Society in 2020, which included 504 responses from 385 centers, elicited considerable variation in clinical practice with regards to obtaining TE MRI in extremely low gestational age infants, not only in the U.S., but also in other parts of the world and especially among different disciplines (i.e., neonatologist, neurologist, radiologist) [32]. Neurologists and radiologists indicated that TE MRI added value to clinical care and decreased parental anxiety, whereas neonatologists were equivocal [32]; as such, there is considerable overlap among many of these sub-specialties in support of TE MRI, especially since one of the main barriers to obtaining TE MRI, i.e. the need for sedation, is currently not a major factor. The survey indicated that in the U.S., the majority of hospitals do not use sedation for brain MRI in infants. Over the last decade multiple studies have demonstrated that TE MRI has a role in predicting neurodevelopmental outcome in premature infants because of its improved ability to assess cerebral development and injury, with MRI outperforming other neuroimaging modalities, as well as clinical and physical exam as a predictive biomarker in these infants [33–35]. Our findings therefore raise concern that an overinflated stated benefit behind this *Choosing Wisely* recommendation (i.e., the overstated potential for annual healthcare savings of $60 million vs. actual of $1.2 million) could have the unintended consequence of suppressing an emerging science regarding TE MRI and care should be taken to find the appropriate balance; this broadly agrees with the recent viewpoint of Paul Fischer MD that “While future research is needed, an agnostic approach right now denying all “routine” brain MRIs at term for children born too early seems premature” [7].

Our study has some notable limitations. First, our cost estimates were calculated using the highest global procedure cost for each study year cycle which may have resulted in an overestimation of cost savings associated with LVS brain MRIs among preterm infants. Second, while our dataset allowed us to readily identify LVS brain MRIs among preterm infants, we were unable to examine other *Top Five* guidelines in newborn medicine with the same level of certainty. Third, it should be noted that the unit of analysis was hospital discharge rather than individual infants which is a potential source of bias, as an infant may have several admissions for the same illness episode, although the likelihood of such an occurrence is considered infrequent. Fourth, another limitation might be that the ICD codes reported do not reflect the actual reason for ordering MRI scans and this could be a source of potential information bias.

On the other hand, our study has several strengths. First, no prior analysis has been conducted to evaluate the cost-savings associated with the *Top Five* list of LVS among neonates. As such, our study offers valuable insight into the financial impact of the *Top Five* list and per Kerr et al. recommendations [3], supports progressive evaluation of effects of other LVS priorities in newborn medicine. Second, we used a national population-based database representative of pediatric discharges throughout a diverse range of NICUs in the U.S. Third, we account for additional expenditure associated with further workup for incidental finding by using sensitivity analyses.

Despite yearly increases in costs, reducing excessive healthcare spending continues to be a challenge within the U.S. as the healthcare landscape transitions from volume-based to value-based services. While elimination of all wasteful spending should be the goal of providers within the healthcare industry, initiatives and campaigns, such as *Choosing Wisely*, are essential to identify practices and policies that contribute significantly to unwarranted spending. Beyond procedure efficacy and cost considerations, however, it will be important to use available national databases that incorporate disease prevalence to evaluate the magnitude of impact both prior to and after guideline implementation. While a $60 million benefit has been advocated regarding the impact of this *Choosing Wisely* recommendation, available data both before and after implementation suggest a much smaller effect. Collaboration across disciplines (e.g., medicine, public health, health economics) will be critical to ensure these guidelines and recommendations are effective in reducing waste within the healthcare system.

## Supporting information

S1 Table List and prevalence of ICD-9/10 primary diagnosis codes for selected preterm infants by MRI indication status. (DOCX)

S2 Table Weighted distribution of low value service and not low value service across cycle years and gestational age. (DOCX)

S1 Fig Relative prevalence of infant gestational age across indicated vs. non-indicated status. (TIF)

## Abbreviations

AAP: American Academy of Pediatrics
AHRQ: Agency for Healthcare Research and Quality
CDC: Centers for Disease Control and Prevention
CMS: Centers Medicaid and Medicare Services
GDP: Gross Domestic Product
HCUP: Healthcare Cost and Utilization Project
ICD-10: International Classification of Diseases, Tenth Revision
ICD-9: International Classification of Diseases, Ninth Revision
KID: Kids’ Inpatient Database
LVS: low value service
MRI: Magnetic resonance imaging
NI: Non-indicated
TE: Term-equivalent
