# Supplementary material for: An assessment of prevalence and expenditure associated with discharge brain MRI in preterm infants
Source: PLoS One. 2021 Mar 5;16(3):e0247857. doi: 10.1371/journal.pone.0247857 (PMC7935297; doi:10.1371/journal.pone.0247857)
Supplement: S1 Table — (DOCX) [file pone.0247857.s001.docx]

**S1 Table.**

| **ICD-9** | **Brief description** | **Non-Indicated (N)** | **Non-Indicated (%)** | **Indicated (N)** | **Indicated (%)** |
| --- | --- | --- | --- | --- | --- |
|  |  |  |  |  |  |
| 765.10 | Preterm infant nec wtnos | 1 | 0.05 | 0 | 0.00 |
| 765.01 | Extreme immatur <500g | 3 | 0.15 | 0 | 0.00 |
| 765.02 | Extreme immatur 500-749g | 32 | 1.62 | 0 | 0.00 |
| 765.03 | Extreme immatur 750-999g | 35 | 1.77 | 0 | 0.00 |
| 765.04 | Extreme immat 1000-1249g | 1 | 0.05 | 0 | 0.00 |
| 765.05 | Extreme immat 1250-1499g | 2 | 0.10 | 0 | 0.00 |
| 765.07 | Extreme immat 1750-1999g | 1 | 0.05 | 0 | 0.00 |
| 765.11 | Preterm nec <500g | 2 | 0.10 | 0 | 0.00 |
| 765.12 | Preterm nec 500-749g | 4 | 0.20 | 0 | 0.00 |
| 765.13 | Preterm nec 750-999g | 11 | 0.56 | 0 | 0.00 |
| 765.14 | Preterm nec 1000-1249g | 29 | 1.47 | 0 | 0.00 |
| 765.15 | Preterm nec 1250-1499g | 16 | 0.81 | 0 | 0.00 |
| 765.16 | Preterm nec 1500-1749g | 17 | 0.86 | 0 | 0.00 |
| 765.17 | Preterm nec 1750-1999g | 13 | 0.66 | 0 | 0.00 |
| 765.18 | Preterm nec 2000-2499g | 19 | 0.96 | 0 | 0.00 |
| 765.19 | Preterm nec 2500+g | 32 | 1.62 | 0 | 0.00 |
| 765.22 | 24 comp weeks gestation | 0 | 0.00 | 1 | 0.40 |
| 765.23 | 25-26 comp wks gestation | 0 | 0.00 | 4 | 1.61 |
| 765.26 | 31-32 comp wks gestation | 0 | 0.00 | 1 | 0.40 |
|  | **Clinically Indicated Disease** |  |  |  |  |
| 005.1 | Botulism food poisoning | 0 | 0.00 | 1 | 0.40 |
| 033.0 | Bordetella pertussis | 1 | 0.05 | 0 | 0.00 |
| 038.0 | Streptococcal septicemia | 1 | 0.05 | 0 | 0.00 |
| 038.9 | Septicemia nos | 3 | 0.15 | 0 | 0.00 |
| 054.3 | Herpetic encephalitis | 0 | 0.00 | 2 | 0.80 |
| 155.2 | Malignant neo liver nos | 1 | 0.05 | 0 | 0.00 |
| 191.5 | Mal neo cereb ventricle | 0 | 0.00 | 1 | 0.40 |
| 191.6 | Mal neo cerebellum nos | 0 | 0.00 | 2 | 0.80 |
| 212.7 | Benign neoplasm heart | 0 | 0.00 | 2 | 0.80 |
| 216.7 | Benign neo skin leg | 1 | 0.05 | 0 | 0.00 |
| 228.01 | Hemangioma skin | 1 | 0.05 | 0 | 0.00 |
| 228.09 | Hemangioma nec | 2 | 0.10 | 0 | 0.00 |
| 243 | Congenital hypothyroidsm | 1 | 0.05 | 0 | 0.00 |
| 244.9 | Hypothyroidism nos | 1 | 0.05 | 0 | 0.00 |
| 253.5 | Diabetes insipidus | 0 | 0.00 | 2 | 0.80 |
| 263.9 | Protein-cal malnutr nos | 1 | 0.05 | 0 | 0.00 |
| 266.1 | Vitamin b6 deficiency | 1 | 0.05 | 0 | 0.00 |
| 277.87 | Dis mitochondrial metab | 0 | 0.00 | 2 | 0.80 |
| 277.9 | Metabolism disorder nos | 1 | 0.05 | 0 | 0.00 |
| 320.2 | Streptococcal meningitis | 3 | 0.15 | 0 | 0.00 |
| 320.82 | Mningts gram-neg bct nec | 1 | 0.05 | 0 | 0.00 |
| 324.0 | Intracranial abscess | 0 | 0.00 | 1 | 0.40 |
| 327.23 | Obstructive sleep apnea | 1 | 0.05 | 0 | 0.00 |
| 327.27 | Cntrl sleep apnea ot dis | 1 | 0.05 | 0 | 0.00 |
| 327.29 | Organic sleep apnea nec | 1 | 0.05 | 0 | 0.00 |
| 331.3 | Communicat hydrocephalus | 0 | 0.00 | 2 | 0.80 |
| 331.4 | Obstructiv hydrocephalus | 0 | 0.00 | 14 | 5.62 |
| 345.10 | Gen cnv epil w/o intr ep | 0 | 0.00 | 1 | 0.40 |
| 345.40 | Psymotr epil w/o int epi | 0 | 0.00 | 1 | 0.40 |
| 345.60 | Inf spasm w/o intr epil | 0 | 0.00 | 5 | 2.01 |
| 345.61 | Inf spasm w intract epil | 0 | 0.00 | 2 | 0.80 |
| 345.90 | Epilep nos w/o intr epil | 0 | 0.00 | 2 | 0.80 |
| 358.8 | Myoneural disorders nec | 0 | 0.00 | 1 | 0.40 |
| 362.21 | Retrolental fibroplasia | 2 | 0.10 | 0 | 0.00 |
| 362.26 | Retinoph prematr.stage 4 | 2 | 0.10 | 0 | 0.00 |
| 377.43 | Optic nerve hypoplasia | 0 | 0.00 | 1 | 0.40 |
| 379.59 | Irregular eye mvmnts nec | 0 | 0.00 | 1 | 0.40 |
| 425.3 | Endocard fibroelastosis | 1 | 0.05 | 0 | 0.00 |
| 425.4 | Prim cardiomyopathy nec | 1 | 0.05 | 0 | 0.00 |
| 427.89 | Cardiac dysrhythmias nec | 1 | 0.05 | 0 | 0.00 |
| 428.0 | Chf nos | 1 | 0.05 | 0 | 0.00 |
| 431 | Intracerebral hemorrhage | 0 | 0.00 | 1 | 0.40 |
| 432.9 | Intracranial hemorr nos | 0 | 0.00 | 1 | 0.40 |
| 434.11 | Crbl emblsm w infrct | 0 | 0.00 | 1 | 0.40 |
| 434.91 | Crbl art ocl nos w infrc | 0 | 0.00 | 1 | 0.40 |
| 446.7 | Takayasu's disease | 1 | 0.05 | 0 | 0.00 |
| 465.9 | Acute uri nos | 1 | 0.05 | 0 | 0.00 |
| 466.11 | Acu broncholitis d/t rsv | 1 | 0.05 | 0 | 0.00 |
| 466.19 | Acu brnchlts d/t oth org | 3 | 0.15 | 0 | 0.00 |
| 478.33 | Vocal paral bilat part | 1 | 0.05 | 0 | 0.00 |
| 507.0 | Food/vomit pneumonitis | 1 | 0.05 | 0 | 0.00 |
| 518.81 | Acute respiratry failure | 5 | 0.25 | 0 | 0.00 |
| 518.84 | Acute & chronc resp fail | 3 | 0.15 | 0 | 0.00 |
| 519.8 | Resp system disease nec | 1 | 0.05 | 0 | 0.00 |
| 524.00 | Unspcf anomaly jaw size | 1 | 0.05 | 0 | 0.00 |
| 530.81 | Esophageal reflux | 10 | 0.51 | 0 | 0.00 |
| 536.8 | Stomach function dis nec | 1 | 0.05 | 0 | 0.00 |
| 550.92 | Bilat inguinal hernia | 1 | 0.05 | 0 | 0.00 |
| 557.0 | Ac vasc insuff intestine | 1 | 0.05 | 0 | 0.00 |
| 558.9 | Noninf gastroenterit nec | 1 | 0.05 | 0 | 0.00 |
| 560.89 | Intestinal obstruct nec | 1 | 0.05 | 0 | 0.00 |
| 579.3 | Intest postop nonabsorb | 1 | 0.05 | 0 | 0.00 |
| 584.9 | Acute kidney failure nos | 1 | 0.05 | 0 | 0.00 |
| 599.0 | Urin tract infection nos | 1 | 0.05 | 0 | 0.00 |
| 682.8 | Cellulitis, site nec | 1 | 0.05 | 0 | 0.00 |
| 741.00 | Spin bif w hydroceph nos | 0 | 0.00 | 3 | 1.20 |
| 741.03 | Spin bif w hydrceph-lumb | 0 | 0.00 | 2 | 0.80 |
| 741.92 | Spina bifida-dorsal | 0 | 0.00 | 1 | 0.40 |
| 741.93 | Spina bifida-lumbar | 0 | 0.00 | 1 | 0.40 |
| 742.0 | Encephalocele | 0 | 0.00 | 3 | 1.20 |
| 742.2 | Reduction deform, brain | 0 | 0.00 | 3 | 1.20 |
| 742.3 | Congenital hydrocephalus | 0 | 0.00 | 18 | 7.23 |
| 742.4 | Brain anomaly nec | 0 | 0.00 | 4 | 1.61 |
| 742.9 | Nervous system anom nos | 0 | 0.00 | 1 | 0.40 |
| 743.03 | Congen cystic eyeball | 0 | 0.00 | 1 | 0.40 |
| 743.33 | Nuclear cataract | 1 | 0.05 | 0 | 0.00 |
| 745.10 | Compl transpos great ves | 2 | 0.10 | 0 | 0.00 |
| 745.11 | Double outlet rt ventric | 1 | 0.05 | 0 | 0.00 |
| 745.3 | Common ventricle | 1 | 0.05 | 0 | 0.00 |
| 745.4 | Ventricular sept defect | 2 | 0.10 | 0 | 0.00 |
| 745.5 | Secundum atrial sept def | 1 | 0.05 | 0 | 0.00 |
| 746.01 | Cong pulmon valv atresia | 1 | 0.05 | 0 | 0.00 |
| 746.02 | Cong pulmon valve stenos | 1 | 0.05 | 0 | 0.00 |
| 746.1 | Cong tricusp atres/sten | 1 | 0.05 | 0 | 0.00 |
| 746.2 | Ebstein's anomaly | 1 | 0.05 | 0 | 0.00 |
| 746.7 | Hypoplas left heart synd | 2 | 0.10 | 0 | 0.00 |
| 746.89 | Cong heart anomaly nec | 2 | 0.10 | 0 | 0.00 |
| 747.0 | Patent ductus arteriosus | 15 | 0.76 | 0 | 0.00 |
| 747.10 | Coarctation of aorta | 3 | 0.15 | 0 | 0.00 |
| 747.21 | Anomalies of aortic arch | 1 | 0.05 | 0 | 0.00 |
| 747.41 | Tot anom pulm ven connec | 1 | 0.05 | 0 | 0.00 |
| 747.81 | Cerebrovascular anomaly | 0 | 0.00 | 1 | 0.40 |
| 747.83 | Persistent fetal circ | 10 | 0.51 | 0 | 0.00 |
| 748.0 | Choanal atresia | 1 | 0.05 | 0 | 0.00 |
| 748.3 | Laryngotrach anomaly nec | 3 | 0.15 | 0 | 0.00 |
| 748.60 | Lung anomaly nos | 2 | 0.10 | 0 | 0.00 |
| 749.21 | Unil cleft palat/lip-com | 2 | 0.10 | 0 | 0.00 |
| 749.22 | Unil cleft palat/lip-inc | 1 | 0.05 | 0 | 0.00 |
| 750.3 | Cong esoph fistula/atres | 4 | 0.20 | 0 | 0.00 |
| 751.1 | Atresia small intestine | 3 | 0.15 | 0 | 0.00 |
| 751.2 | Atresia large intestine | 2 | 0.10 | 0 | 0.00 |
| 751.3 | Hirschsprung's disease | 1 | 0.05 | 0 | 0.00 |
| 751.5 | Intestinal anomaly nec | 3 | 0.15 | 0 | 0.00 |
| 753.14 | Polycyst kid-autosom rec | 1 | 0.05 | 0 | 0.00 |
| 753.15 | Renal dysplasia | 1 | 0.05 | 0 | 0.00 |
| 753.6 | Congen urethral stenosis | 1 | 0.05 | 0 | 0.00 |
| 754.89 | Nonteratogenic anom nec | 2 | 0.10 | 0 | 0.00 |
| 755.55 | Acrocephalosyndactyly | 0 | 0.00 | 2 | 0.80 |
| 756.0 | Anomal skull/face bones | 0 | 0.00 | 6 | 2.41 |
| 756.13 | Cong absence of vertebra | 1 | 0.05 | 0 | 0.00 |
| 756.17 | Spina bifida occulta | 0 | 0.00 | 1 | 0.40 |
| 756.6 | Anomalies of diaphragm | 1 | 0.05 | 0 | 0.00 |
| 756.73 | Gastroschisis | 1 | 0.05 | 0 | 0.00 |
| 756.79 | Congn anoml abd wall nec | 1 | 0.05 | 0 | 0.00 |
| 757.39 | Skin anomaly nec | 1 | 0.05 | 0 | 0.00 |
| 758.33 | Microdeletions nec | 1 | 0.05 | 0 | 0.00 |
| 758.5 | Autosomal anomalies nec | 3 | 0.15 | 0 | 0.00 |
| 759.2 | Endocrine anomaly nec | 1 | 0.05 | 0 | 0.00 |
| 759.3 | Situs inversus | 1 | 0.05 | 0 | 0.00 |
| 759.5 | Tuberous sclerosis | 0 | 0.00 | 1 | 0.40 |
| 759.6 | Hamartoses nec | 1 | 0.05 | 0 | 0.00 |
| 759.81 | Prader-willi syndrome | 4 | 0.20 | 0 | 0.00 |
| 759.89 | Specfied cong anomal nec | 0 | 0.00 | 5 | 2.01 |
| 760.5 | Maternal injury aff nb | 1 | 0.05 | 0 | 0.00 |
| 761.3 | Polyhydramnios aff nb | 1 | 0.05 | 0 | 0.00 |
| 761.7 | Antepart malpres aff nb | 1 | 0.05 | 0 | 0.00 |
| 762.8 | Abn amnion nec aff nb | 1 | 0.05 | 0 | 0.00 |
| 764.05 | Lt-for-dates 1250-1499g | 1 | 0.05 | 0 | 0.00 |
| 764.92 | Fet growth ret 500-749g | 1 | 0.05 | 0 | 0.00 |
| 764.97 | Fet grwth ret 1750-1999g | 2 | 0.10 | 0 | 0.00 |
| 764.98 | Fet grwth ret 2000-2499g | 1 | 0.05 | 0 | 0.00 |
| 767.0 | Cerebral hem at birth | 0 | 0.00 | 7 | 2.81 |
| 767.19 | Injuries to scalp nec | 2 | 0.10 | 0 | 0.00 |
| 767.3 | Bone injury nec at birth | 2 | 0.10 | 0 | 0.00 |
| 767.8 | Birth trauma nec | 0 | 0 | 0 | 0.00 |
| 768.5 | Severe birth asphyxia | 0 | 0 | 5 | 2.01 |
| 768.7 | Hypoxic-ischemic enceph# | 0 | 0 | 10 | 4.02 |
| 768.70 | Hypoxc-ischem enceph nos | 0 | 0 | 8 | 3.21 |
| 768.71 | Mild hypox-ischem enceph | 0 | 0 | 1 | 0.40 |
| 768.72 | Mod hypox-ischem enceph | 0 | 0 | 2 | 0.80 |
| 768.73 | Sev hypox-ischem enceph | 0 | 0 | 2 | 0.80 |
| 768.9 | Birth asphyxia nos | 0 | 0 | 2 | 0.80 |
| 769 | Respiratory distress syn | 105 | 5.31 | 0 | 0 |
| 770.0 | Congenital pneumonia | 1 | 0.05 | 0 | 0 |
| 770.11 | Meconium asp wo resp sym | 1 | 0.05 | 0 | 0 |
| 770.2 | Nb interstit emphysema | 4 | 0.20 | 0 | 0 |
| 770.3 | Nb pulmonary hemorrhage | 2 | 0.10 | 0 | 0 |
| 770.6 | Nb transitory tachypnea | 5 | 0.25 | 0 | 0 |
| 770.7 | Perinatal chr resp dis | 12 | 0.61 | 0 | 0 |
| 770.81 | Primary apnea of newborn | 25 | 1.26 | 0 | 0 |
| 770.82 | Other apnea of newborn | 2 | 0.10 | 6 | 2.41 |
| 770.84 | Resp failure of newborn | 2 | 0.10 | 0 | 0.00 |
| 770.89 | Resp prob after brth nec | 9 | 0.46 | 0 | 0.00 |
| 770.9 | Nb respiratory cond nos | 1 | 0.05 | 0 | 0.00 |
| 771.1 | Cong cytomegalovirus inf | 0 | 0.00 | 3 | 1.20 |
| 771.2 | Congenital infec nec | 4 | 0.20 | 0 | 0.00 |
| 771.81 | Nb septicemia [sepsis] | 25 | 1.26 | 0 | 0.00 |
| 771.82 | Nb urinary tract infectn | 2 | 0.10 | 0 | 0.00 |
| 771.89 | Perinatal infection nec | 9 | 0.46 | 0 | 0.00 |
| 772.10 | Nb intraven hem nos | 4 | 0.20 | 0 | 0.00 |
| 772.13 | Nb intravn hem,grade iii | 10 | 0.51 | 0 | 0.00 |
| 772.14 | Nb intraven hem,grade iv | 0 | 0.00 | 9 | 3.61 |
| 772.2 | Nb subarachnoid hemorr | 0 | 0.00 | 2 | 0.80 |
| 772.5 | Nb adrenal hemorrhage | 1 | 0.05 | 0 | 0.00 |
| 773.0 | Nb hemolyt dis:rh isoimm | 1 | 0.05 | 0 | 0.00 |
| 774.2 | Neonat jaund preterm del | 3 | 0.15 | 0 | 0.00 |
| 774.4 | Fetal/neonatal hepatitis | 1 | 0.05 | 0 | 0.00 |
| 775.0 | Infant diabet mother syn | 2 | 0.10 | 0 | 0.00 |
| 775.3 | Neonatal thyrotoxicosis | 1 | 0.05 | 0 | 0.00 |
| 775.5 | Neonatal dehydration | 2 | 0.10 | 0 | 0.00 |
| 775.6 | Neonatal hypoglycemia | 6 | 0.30 | 0 | 0.00 |
| 775.7 | Late metab acidosis nb | 1 | 0.05 | 0 | 0.00 |
| 775.8 | Transient met dis nb nec# | 2 | 0.10 | 0 | 0.00 |
| 775.81 | Nb acidosis nec | 2 | 0.10 | 0 | 0.00 |
| 775.89 | Neonat endo/met dis nec | 0 | 0.00 | 1 | 0.40 |
| 776.1 | Neonatal thrombocytopen | 0 | 0.00 | 2 | 0.80 |
| 776.5 | Congenital anemia | 1 | 0.05 | 0 | 0.00 |
| 777.1 | Meconium obstruction | 4 | 0.20 | 0 | 0.00 |
| 777.5 | Necrot enterocolitis nb# | 4 | 0.20 | 0 | 0.00 |
| 777.50 | Nec enterocoltis nb nos | 4 | 0.20 | 0 | 0.00 |
| 777.52 | Stg ii nec enterocol nb | 2 | 0.10 | 0 | 0.00 |
| 777.53 | Stg iii nec enterocol nb | 2 | 0.10 | 0 | 0.00 |
| 777.6 | Perinatal intest perfor | 2 | 0.10 | 0 | 0.00 |
| 777.8 | Perinat gi sys dis nec | 1 | 0.05 | 0 | 0.00 |
| 777.9 | Perinat gi sys dis nos | 1 | 0.05 | 0 | 0.00 |
| 778.0 | Hydrops fetalis no isoim | 2 | 0.10 | 0 | 0.00 |
| 778.3 | Nb hypothermia nec | 3 | 0.15 | 0 | 0.00 |
| 779.0 | Convulsions in newborn | 0 | 0.00 | 19 | 7.63 |
| 779.3 | Nb feeding problems# | 5 | 0.25 | 0 | 0.00 |
| 779.31 | Nb feeding problems | 3 | 0.15 | 0 | 0.00 |
| 779.5 | Nb drug withdrawal syndr | 2 | 0.10 | 0 | 0.00 |
| 779.7 | Perivent leukomalacia | 0 | 0.00 | 1 | 0.40 |
| 779.89 | Perinatal condition nec | 0 | 0.00 | 35 | 14.06 |
| 780.31 | Febrile convulsions nos | 2 | 0.10 | 0 | 0.00 |
| 780.32 | Complx febrile convulsns | 1 | 0.05 | 0 | 0.00 |
| 780.39 | Convulsions nec | 0 | 0.00 | 12 | 4.82 |
| 781.3 | Lack of coordination | 0 | 0.00 | 1 | 0.40 |
| 782.5 | Cyanosis | 1 | 0.05 | 0 | 0.00 |
| 783.3 | Feeding problem | 3 | 0.15 | 0 | 0.00 |
| 783.40 | Lack norm physio dev nos | 2 | 0.10 | 0 | 0.00 |
| 783.41 | Failure to thrive-child | 4 | 0.20 | 0 | 0.00 |
| 786.03 | Apnea | 2 | 0.10 | 0 | 0.00 |
| 786.1 | Stridor | 1 | 0.05 | 0 | 0.00 |
| 787.21 | Dysphagia, oral phase | 1 | 0.05 | 0 | 0.00 |
| 789.59 | Ascites nec | 1 | 0.05 | 0 | 0.00 |
| 799.82 | Appar life threat-infant | 1 | 0.05 | 0 | 0.00 |
| 801.31 | Cl skul base fx w/o coma | 0 | 0.00 | 1 | 0.40 |
| 852.01 | Subarachnoid hem-no coma | 0 | 0.00 | 1 | 0.40 |
| 852.20 | Traumatic subdural hem | 0 | 0.00 | 1 | 0.40 |
| 852.21 | Subdural hem w/o coma | 0 | 0.00 | 3 | 1.20 |
| 994.7 | Asphyxiation/strangulat | 0 | 0.00 | 1 | 0.40 |
| 995.50 | Child abuse nos | 0 | 0.00 | 1 | 0.40 |
| 995.54 | Child physical abuse | 0 | 0.00 | 4 | 1.61 |
| 995.55 | Shaken infant syndrome | 0 | 0.00 | 1 | 0.40 |
| 996.2 | Malfun neuro device/graf | 0 | 0.00 | 1 | 0.40 |
| 996.74 | Comp-oth vasc dev/graft | 1 | 0.05 | 0 | 0.00 |
| 997.39 | Respiratory comp nec | 1 | 0.05 | 0 | 0.00 |
| V30.00 | Single lb in-hosp w/o cs | 358 | 18.10 | 0 | 0.00 |
| V30.01 | Single lb in-hosp w cs | 663 | 33.52 | 0 | 0.00 |
| V30.1 | Singl livebrn-before adm | 12 | 0.61 | 0 | 0.00 |
| V31.00 | Twin-mate lb-hosp w/o cs | 28 | 1.42 | 0 | 0.00 |
| V31.01 | Twin-mate lb-in hos w cs | 194 | 9.81 | 0 | 0.00 |
| V31.1 | Twin, mate lb-before adm | 3 | 0.15 | 0 | 0.00 |
| V32.00 | Twin-mate sb-hosp w/o cs | 2 | 0.10 | 0 | 0.00 |
| V32.01 | Twin-mate sb-hosp w cs | 9 | 0.46 | 0 | 0.00 |
| V33.00 | Twin-nos-in hosp w/o cs | 1 | 0.05 | 0 | 0.00 |
| V34.00 | Oth mult lb-hosp w/o cs | 1 | 0.05 | 0 | 0.00 |
| V34.01 | Oth mult lb-in hosp w cs | 23 | 1.16 | 0 | 0.00 |
| V36.00 | Mult lb/sb-in hos w/o cs | 2 | 0.10 | 0 | 0.00 |
| V58.89 | Other specfied aftercare | 1 | 0.05 | 0 | 0.00 |
| V63.8 | No med facilities nec | 1 | 0.05 | 0 | 0.00 |
| V71.89 | Observ-suspect cond nec | 1 | 0.05 | 0 | 0.00 |

| **ICD-10 Code** | **Brief Description** | **Non-Indicated (N)** | **Non-Indicated (%)** | **Indicated (N)** | **Indicated (%)** |
| --- | --- | --- | --- | --- | --- |
|  |  |  |  |  |  |
| P07.22 | Extreme immaturity of NB, gestatnl age 23 completed weeks | 0 | 0.00 | 1 | 0.47 |
| P07.33 | Preterm newborn, gestational age 30 completed weeks | 0 | 0.00 | 1 | 0.47 |
| P07.35 | Preterm newborn, gestational age 32 completed weeks | 0 | 0.00 | 2 | 0.93 |
| P07.36 | Preterm newborn, gestational age 33 completed weeks | 0 | 0.00 | 2 | 0.93 |
| P07.37 | Preterm newborn, gestational age 34 completed weeks | 0 | 0.00 | 3 | 1.40 |
| P07.38 | Preterm newborn, gestational age 35 completed weeks | 0 | 0.00 | 3 | 1.40 |
| P07.39 | Preterm newborn, gestational age 36 completed weeks | 0 | 0.00 | 2 | 0.93 |
|  | **Clinically Indicated Disease** |  |  |  |  |
| A40.3 | Sepsis due to Streptococcus pneumoniae | 1 | 0.47 | 0 | 0.00 |
| A41.51 | Sepsis due to Escherichia coli [E. coli] | 1 | 0.47 | 0 | 0.00 |
| A41.9 | Sepsis, unspecified organism | 3 | 1.40 | 0 | 0.00 |
| B00.7 | Disseminated herpesviral disease | 1 | 0.47 | 0 | 0.00 |
| B00.9 | Herpesviral infection, unspecified | 1 | 0.47 | 0 | 0.00 |
| D18.09 | Hemangioma of other sites | 0 | 0.00 | 1 | 0.47 |
| D81.81 | Biotinidase deficiency | 1 | 0.47 | 0 | 0.00 |
| E23.2 | Diabetes insipidus | 0 | 0.00 | 1 | 0.47 |
| E44.0 | Moderate protein-calorie malnutrition | 1 | 0.47 | 0 | 0.00 |
| E63.9 | Nutritional deficiency, unspecified | 1 | 0.47 | 0 | 0.00 |
| E75.29 | Other sphingolipidosis | 0 | 0.00 | 1 | 0.47 |
| E83.09 | Other disorders of copper metabolism | 0 | 0.00 | 1 | 0.47 |
| G00.2 | Streptococcal meningitis | 1 | 0.47 | 0 | 0.00 |
| G00.9 | Bacterial meningitis, unspecified | 1 | 0.47 | 0 | 0.00 |
| G40.40 | Oth generalized epilepsy, not intractable, w/o stat epi | 0 | 0.00 | 1 | 0.47 |
| G40.82 | Epileptic spasms, not intractable, w/o status epilepticus | 0 | 0.00 | 5 | 2.33 |
| G40.90 | Epilepsy, unsp, not intractable, with status epilepticus | 0 | 0.00 | 2 | 0.93 |
| G40.90 | Epilepsy, unsp, not intractable, without status epilepticus | 0 | 0.00 | 2 | 0.93 |
| G47.33 | Obstructive sleep apnea (adult) (pediatric) | 1 | 0.47 | 0 | 0.00 |
| G91.8 | Other hydrocephalus | 0 | 0.00 | 1 | 0.47 |
| G91.9 | Hydrocephalus, unspecified | 0 | 0.00 | 1 | 0.47 |
| G93.1 | Anoxic brain damage, not elsewhere classified | 0 | 0.00 | 1 | 0.47 |
| H35.14 | Retinopathy of prematurity, stage 3, bilateral | 2 | 0.93 | 0 | 0.00 |
| H55.89 | Other irregular eye movements | 0 | 0.00 | 1 | 0.47 |
| I62.00 | Nontraumatic subdural hemorrhage, unspecified | 0 | 0.00 | 1 | 0.47 |
| I62.01 | Nontraumatic acute subdural hemorrhage | 0 | 0.00 | 1 | 0.47 |
| I62.03 | Nontraumatic chronic subdural hemorrhage | 0 | 0.00 | 1 | 0.47 |
| I82.3 | Embolism and thrombosis of renal vein | 1 | 0.47 | 0 | 0.00 |
| J14 | Pneumonia due to Hemophilus influenzae | 1 | 0.47 | 0 | 0.00 |
| J15.1 | Pneumonia due to Pseudomonas | 1 | 0.47 | 0 | 0.00 |
| J21.0 | Acute bronchiolitis due to respiratory syncytial virus | 1 | 0.47 | 0 | 0.00 |
| J21.1 | Acute bronchiolitis due to human metapneumovirus | 1 | 0.47 | 0 | 0.00 |
| J21.8 | Acute bronchiolitis due to other specified organisms | 1 | 0.47 | 0 | 0.00 |
| J84.84 | Pulmonary interstitial glycogenosis | 1 | 0.47 | 0 | 0.00 |
| J95.02 | Infection of tracheostomy stoma | 1 | 0.47 | 0 | 0.00 |
| J96.01 | Acute respiratory failure with hypoxia | 0 | 0.00 | 1 | 0.47 |
| J96.10 | Chronic respiratory failure, unsp w hypoxia or hypercapnia | 0 | 0.00 | 1 | 0.47 |
| J96.21 | Acute and chronic respiratory failure with hypoxia | 0 | 0.00 | 1 | 0.47 |
| J96.22 | Acute and chronic respiratory failure with hypercapnia | 1 | 0.47 | 0 | 0.00 |
| K21.9 | Gastro-esophageal reflux disease without esophagitis | 2 | 0.93 | 0 | 0.00 |
| K90.49 | Malabsorption due to intolerance, not elsewhere classified | 1 | 0.47 | 0 | 0.00 |
| N04.9 | Nephrotic syndrome with unspecified morphologic changes | 1 | 0.47 | 0 | 0.00 |
| P07.02 | Extremely low birth weight newborn, 500-749 grams | 10 | 4.65 | 0 | 0.00 |
| P07.03 | Extremely low birth weight newborn, 750-999 grams | 0 | 0.00 | 11 | 5.12 |
| P07.14 | Other low birth weight newborn, 1000-1249 grams | 0 | 0.00 | 9 | 4.19 |
| P07.15 | Other low birth weight newborn, 1250-1499 grams | 0 | 0.00 | 2 | 0.93 |
| P07.16 | Other low birth weight newborn, 1500-1749 grams | 0 | 0.00 | 3 | 1.40 |
| P07.17 | Other low birth weight newborn, 1750-1999 grams | 0 | 0.00 | 4 | 1.86 |
| P07.18 | Other low birth weight newborn, 2000-2499 grams | 5 | 2.33 | 0 | 0.00 |
|  |  |  |  |  |  |
| P22.0 | Respiratory distress syndrome of newborn | 13 | 6.05 | 0 | 0.00 |
| P22.9 | Respiratory distress of newborn, unspecified | 2 | 0.93 | 0 | 0.00 |
| P24.31 | Neonatal aspirat of milk and regurgitated food w resp symp | 0 | 0.00 | 1 | 0.47 |
| P27.1 | Bronchopulmonary dysplasia origin in the perinatal period | 7 | 3.26 | 0 | 0.00 |
| P28.4 | Other apnea of newborn | 3 | 1.40 | 0 | 0.00 |
| P28.5 | Respiratory failure of newborn | 17 | 7.91 | 0 | 0.00 |
| P29.3 | Persistent fetal circulation | 1 | 0.47 | 0 | 0.00 |
| P35.1 | Congenital cytomegalovirus infection | 0 | 0.00 | 2 | 0.93 |
| P35.2 | Congenital herpesviral [herpes simplex] infection | 3 | 1.40 | 0 | 0.00 |
| P36.0 | Sepsis of newborn due to streptococcus, group B | 1 | 0.47 | 0 | 0.00 |
| P36.8 | Other bacterial sepsis of newborn | 2 | 0.93 | 0 | 0.00 |
| P36.9 | Bacterial sepsis of newborn, unspecified | 11 | 5.12 | 0 | 0.00 |
| P39.8 | Other specified infections specific to the perinatal period | 1 | 0.47 | 0 | 0.00 |
| P52.21 | Intraventricular hemorrhage, grade 3, of newborn | 0 | 0.00 | 2 | 0.93 |
| P52.22 | Intraventricular hemorrhage, grade 4, of newborn | 5 | 2.33 | 0 | 0.00 |
| P59.0 | Neonatal jaundice associated with preterm delivery | 2 | 0.93 | 0 | 0.00 |
| P59.9 | Neonatal jaundice, unspecified | 1 | 0.47 | 0 | 0.00 |
| P70.4 | Other neonatal hypoglycemia | 1 | 0.47 | 0 | 0.00 |
| P76.1 | Transitory ileus of newborn | 1 | 0.47 | 0 | 0.00 |
| P77.2 | Stage 2 necrotizing enterocolitis in newborn | 0 | 0.00 | 1 | 0.47 |
| P77.3 | Stage 3 necrotizing enterocolitis in newborn | 0 | 0.00 | 1 | 0.47 |
| P78.83 | Newborn esophageal reflux | 2 | 0.93 | 0 | 0.00 |
| P83.8 | Other specified conditions of integument specific to newborn | 1 | 0.47 | 0 | 0.00 |
| P90 | Convulsions of newborn | 0 | 0.00 | 6 | 2.79 |
| P91.60 | Hypoxic ischemic encephalopathy [HIE], unspecified | 0 | 0.00 | 8 | 3.72 |
| P91.61 | Mild hypoxic ischemic encephalopathy [HIE] | 0 | 0.00 | 1 | 0.47 |
| P91.62 | Moderate hypoxic ischemic encephalopathy [HIE] | 0 | 0.00 | 3 | 1.40 |
| P91.63 | Severe hypoxic ischemic encephalopathy [HIE] | 0 | 0.00 | 8 | 3.72 |
| P92.9 | Feeding problem of newborn, unspecified | 1 | 0.47 | 0 | 0.00 |
| P96.89 | Oth conditions originating in the perinatal period | 5 | 2.33 | 0 | 0.00 |
| Q01.2 | Occipital encephalocele | 0 | 0.00 | 1 | 0.47 |
| Q02 | Microcephaly | 0 | 0.00 | 1 | 0.47 |
| Q03.0 | Malformations of aqueduct of Sylvius | 0 | 0.00 | 1 | 0.47 |
| Q03.8 | Other congenital hydrocephalus | 0 | 0.00 | 3 | 1.40 |
| Q03.9 | Congenital hydrocephalus, unspecified | 0 | 0.00 | 2 | 0.93 |
| Q04.0 | Congenital malformations of corpus callosum | 0 | 0.00 | 1 | 0.47 |
| Q04.3 | Other reduction deformities of brain | 0 | 0.00 | 1 | 0.47 |
| Q04.6 | Congenital cerebral cysts | 0 | 0.00 | 1 | 0.47 |
| Q04.8 | Other specified congenital malformations of brain | 0 | 0.00 | 2 | 0.93 |
| Q05.2 | Lumbar spina bifida with hydrocephalus | 0 | 0.00 | 1 | 0.47 |
| Q05.5 | Cervical spina bifida without hydrocephalus | 0 | 0.00 | 1 | 0.47 |
| Q07.03 | Arnold-Chiari syndrome with spina bifida and hydrocephalus | 0 | 0.00 | 3 | 1.40 |
| Q20.4 | Double inlet ventricle | 1 | 0.47 | 0 | 0.00 |
| Q21.0 | Ventricular septal defect | 1 | 0.47 | 0 | 0.00 |
| Q21.3 | Tetralogy of Fallot | 1 | 0.47 | 0 | 0.00 |
| Q22.8 | Other congenital malformations of tricuspid valve | 1 | 0.47 | 0 | 0.00 |
| Q25.0 | Patent ductus arteriosus | 4 | 1.86 | 0 | 0.00 |
| Q25.5 | Atresia of pulmonary artery | 1 | 0.47 | 0 | 0.00 |
| Q26.2 | Total anomalous pulmonary venous connection | 1 | 0.47 | 0 | 0.00 |
| Q28.2 | Arteriovenous malformation of cerebral vessels | 0 | 0.00 | 1 | 0.47 |
| Q31.5 | Congenital laryngomalacia | 2 | 0.93 | 0 | 0.00 |
| Q32.0 | Congenital tracheomalacia | 1 | 0.47 | 0 | 0.00 |
| Q42.3 | Congenital absence, atresia and stenosis of anus w/o fistula | 1 | 0.47 | 0 | 0.00 |
| Q44.2 | Atresia of bile ducts | 1 | 0.47 | 0 | 0.00 |
| Q61.4 | Renal dysplasia | 1 | 0.47 | 0 | 0.00 |
| Q67.2 | Dolichocephaly | 0 | 0.00 | 1 | 0.47 |
| Q87.0 | Congen malform syndromes predom affecting facial appearance | 1 | 0.47 | 0 | 0.00 |
| Q87.2 | Congenital malformation syndromes predom involving limbs | 1 | 0.47 | 0 | 0.00 |
| Q89.8 | Other specified congenital malformations | 2 | 0.93 | 0 | 0.00 |
| Q96.0 | Karyotype 45, X | 1 | 0.47 | 0 | 0.00 |
| R53.83 | Other fatigue | 1 | 0.47 | 0 | 0.00 |
| R56.01 | Complex febrile convulsions | 1 | 0.47 | 0 | 0.00 |
| R56.9 | Unspecified convulsions | 0 | 0.00 | 3 | 1.40 |
| R63.3 | Feeding difficulties | 1 | 0.47 | 0 | 0.00 |
| R68.13 | Apparent life threatening event in infant (ALTE) | 1 | 0.47 | 0 | 0.00 |
| T76.12XA | Child physical abuse, suspected, initial encounter | 0 | 0.00 | 3 | 1.40 |
| T85.01XA | Breakdown of ventricular intracranial shunt, init | 0 | 0.00 | 1 | 0.47 |
| T85.79 | Infect/inflm reaction due to oth int prosth dev/grft, init | 1 | 0.47 | 0 | 0.00 |
| Z38.00 | Single liveborn infant, delivered vaginally | 46 | 21.40 | 0 | 0.00 |
| Z38.01 | Single liveborn infant, delivered by cesarean | 0 | 0.00 | 86 | 40.00 |
| Z38.30 | Twin liveborn infant, delivered vaginally | 0 | 0.00 | 2 | 0.93 |
| Z38.31 | Twin liveborn infant, delivered by cesarean | 18 | 8.37 | 0 | 0.00 |
| Z38.62 | Triplet liveborn infant, delivered by cesarean | 1 | 0.47 | 0 | 0.00 |
| Z43.2 | Encounter for attention to ileostomy | 1 | 0.47 | 0 | 0.00 |
