# Supplementary material for: An assessment of prevalence and expenditure associated with discharge brain MRI in preterm infants
Source: PLoS One. 2021 Mar 5;16(3):e0247857. doi: 10.1371/journal.pone.0247857 (PMC7935297; doi:10.1371/journal.pone.0247857)
Supplement: S2 Table — (DOCX) [file pone.0247857.s002.docx]

S2 Table. Weighted distribution^1^ of Low Value Service and Not Low Value Service across Cycle Years and Gestational Age.

|  | **Gestational Age <35 weeks** | | **Gestational Age 35-36 weeks** | |
| --- | --- | --- | --- | --- |
|  | **Low Value Service** | **Not Low Value Service** | **Low Value Service** | **Not Low Value Service** |
| **Cycle Year** | **N (%)** | **N (%)** | **N (%)** | **N (%)** |
| **2006** | 516.6 (89.5) | 60.7 (10.5) | 188.5 (74.8) | 63.5 (25.2) |
| **2009** | 536.7 (87.9) | 73.9 (12.1) | 215.7 (82.3) | 45.0 (17.3) |
| **2012** | 588.4 (90.6) | 60.9 (9.4) | 241.7 (83.5) | 47.7 (16.5) |
| **2016** | 217.8 (52.6) | 195 (47.3) | 67.2 (44.9) | 82.4 (55.1) |
| **Total** | 1859 (82.6) | 390.7 (17.4) | 713.1 (74.9) | 238.6 (25.1) |

^1^Number and percentages weighted to total discharged population in AHA universe.
