# Supplementary figures and images for: An assessment of prevalence and expenditure associated with discharge brain MRI in preterm infants
Source: PLoS One. 2021 Mar 5;16(3):e0247857. doi: 10.1371/journal.pone.0247857 (PMC7935297; doi:10.1371/journal.pone.0247857)

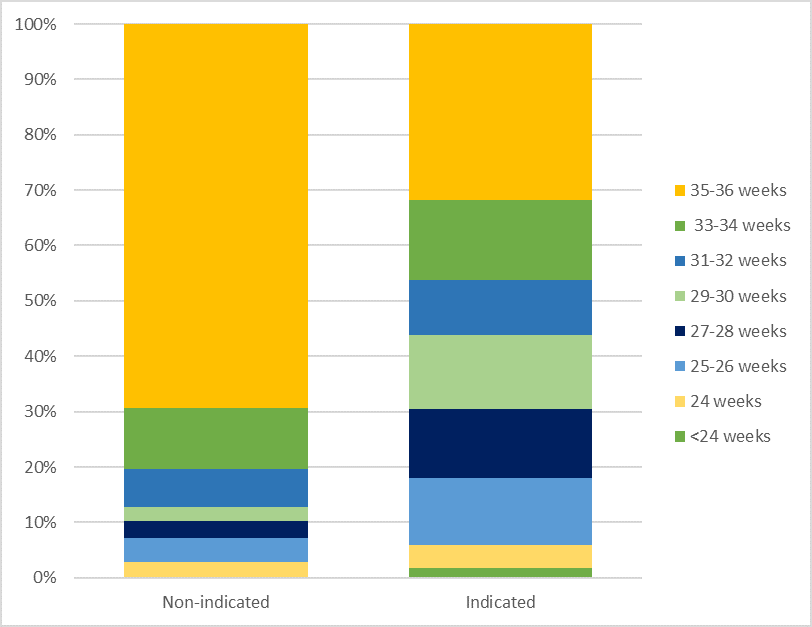

Supplement: S1 Fig — (TIF) [file pone.0247857.s003.tif]
